# Cytokine Profile in a Cohort of Healthy Blood Donors Carrying Polymorphisms in Genes Encoding the NLRP3 Inflammasome

**DOI:** 10.1371/journal.pone.0075457

**Published:** 2013-10-03

**Authors:** Berolla Sahdo, Karin Fransén, Berhane Asfaw Idosa, Per Eriksson, Bo Söderquist, Anne Kelly, Eva Särndahl

**Affiliations:** 1 Department of Clinical Medicine, School of Health & Medical Sciences, Örebro University, Örebro, Sweden; 2 Division of Rheumatology, Department of Clinical and Experimental Medicine, Faculty of Health Sciences, Linköping University, Linköping, Sweden; 3 Department of Laboratory Medicine, Örebro University Hospital, Örebro, Sweden; 4 Faculty of Medicine and Health, Örebro University, Örebro, Sweden; INSERM, France

## Abstract

**Background:**

The NLRP3 inflammasome has been recognized as one of the key components of the innate immunity by sensing a diversity of insults. Inflammasome activation results in the maturation of the pro-inflammatory cytokines interleukin (IL)-1β and IL-18. Increased production of IL-1β is found in patients with gain-of-function polymorphisms in genes encoding the NLRP3 inflammasome. Since approximately 5% of the Swedish population are heterozygote carriers of these combined gene variants, their impact on inflammasome status and a relationship on disease development is therefore highly relevant to study. The present study investigates levels of inflammasome-produced cytokines as a measure of inflammasome activation in healthy individuals carrying Q705K polymorphism in the *NLRP3* gene combined with C10X in the *CARD8* gene.

**Materials and Methods:**

Genotyping of 1006 healthy blood donors was performed for the polymorphisms Q705K in the *NLRP3* and C10X in the *CARD8* genes. IL-1β, IL-18, IL-33, as well as a number of other pro-inflammatory cytokines, were analyzed by Luminex or ELISA in plasma from individuals carrying the polymorphisms and in age and gender matched non-carrier controls.

**Results & Discussion:**

The prevalence of the polymorphisms was in line with previous studies. Plasma levels of IL-1β and IL-33 were elevated among carriers of combined Q705K+C10X polymorphisms compared to controls, whereas no difference was found for IL-18 and the other cytokines measured. Moreover, carriers of C10X or Q705K *per* *se* had similar plasma levels of IL-1β as non-carriers. These data suggest that the combined polymorphisms create inflammasomes with increased basal activation state, which might provide a more favourable innate immune response. In spite of this, it could also represent the mechanisms by which the inflammatory loop is triggered into a long-term inflammatory phenotype.

## Introduction

The NLRP3 inflammasome (formerly known as NALP3 or cryopyrin) has been recognized as one of the key components of innate immunity by sensing microbial ligands, endogenous danger signals and crystalline substances in the cytosol. Upon activation, the sensor protein NLRP3 assembles together with the adaptor protein ASC and pro-caspase-1 into a multi-protein complex called the ‘inflammasome’ (reviewed in [1,2]:). The formation of the inflammasome leads to the auto-proteolytic maturation of caspase-1, which subsequently results in maturation and extracellular release of the pro-inflammatory cytokines interleukin (IL)-1β and IL-18 [3]. The adaptor protein CARD8 (also known as TUCAN) has been suggested to regulate IL-1β secretion via interaction to caspase-1 and/or by inhibiting NFκB-mediated synthesis of pro-IL-1β [4,5]. Recently, IL-33 was suggested to be a substrate for caspase-1, but the processing seems to results in its inactivation [6].

Gain-of-function mutations in the *NLRP3* gene (NCBI reference: NM_004895.3) lead to a constitutive activate NLRP3 protein, resulting in an uncontrolled production of IL-1β. These mutations have been implicated in hereditary inflammatory diseases, often grouped under Cryopyrin-associated periodic syndromes (CAPS) [7,8]. So far, almost all of these disease-causing mutations have been reported in exon 3 of the *NLRP3* gene; suggesting the presence of functionally important sites in this region. We and others have reported patients with CAPS-like symptoms like fever, fatigue, and arthralgia, but who lack the classical manifestations that characterize CAPS [9-13]. These atypical CAPS cases display considerable clinical variability and their phenotype has been suggested to be related to mutations [9-11] or to common polymorphisms [12,13] in the genes encoding the NLRP3 inflammasome. Q705K (rs 35829419) in exon 3 of the *NLRP3* gene (reported as Q703K in the Infevers database [14]) and C10X (rs 2043211) in *CARD8* (NCBI reference: NM_014959) are two such polymorphisms that, *per se* or combined, have been implicated with increased risk of chronic inflammation [15-19]. Recent data from *in vitro* studies reveal that the Q705K is a gain-of-function phenotype associated with increased production of IL-1β [20], whereas C10X results in a truncated non-functional protein [21] and thus the loss of CARD8-mediated inhibition of caspase-1, i.e. both polymorphisms may thereby create a more susceptible inflammasome. The abundance of these polymorphisms in the general population makes it highly relevant to investigate the functional state of the NLRP3 inflammasome in healthy individuals and relate the findings to the gene polymorphisms. In the present study, a cohort of 1006 healthy blood/plasma donors was screened for Q705K in the *NLRP3* and C10X in the *CARD8* genes, and the combined presence of both these polymorphisms was found in 7.3% of the individuals. The plasma levels of IL-1β, IL-18 and IL-33 were determined in this sub-cohort together with age and gender matched non-carrier controls.

## Materials and Methods

### Study subjects and collection of blood

Peripheral whole blood samples were collected in EDTA tubes (K_2_ EDTA Vacuette®, Greiner Bio-one) from a total of 1006 healthy blood and plasma donors (63% male, 37% female; mean age: 42 years, range: 19-67 years) at the Department of Transfusion Medicine, Örebro University Hospital, Sweden. For each blood donor, whole blood was aliquoted for DNA analysis, whereas the remaining blood was centrifuged at 2000xg for 10 min for collection of plasma for cytokine detection. The aliquoted blood and plasma were stored in -80°C.

**Figure 1:**
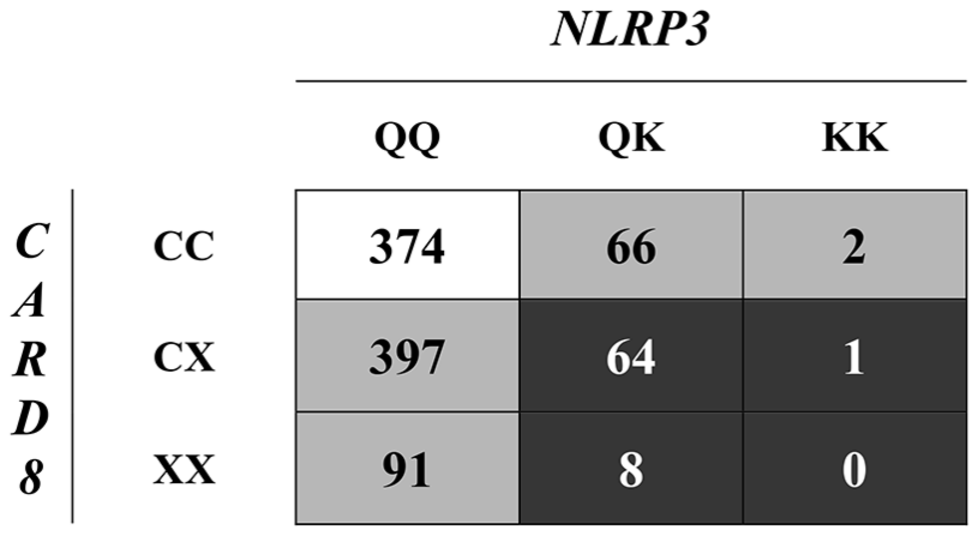
Sub-set selection of Cases and Controls. Genetic analysis was performed in a cohort of 1003 healthy blood/plasma donors. Seventy-three individuals (i.e. 7.3% of the cohort) carried the combined polymorphisms of *NLRP3* (Q705K) and *CARD8* (C10X) (black boxes), and were selected and referred to as combined cases. As controls, 147 age and gender matched wild-types (white box) were selected. In addition, samples from individual being homozygote (n=47) or heterozygote (n=50) for C10X, or homozygote (n=2) or heterozygote (n=50) for Q705K were referred to as cases (grey boxes). As controls, 100 age and gender matched wild-types (white box) were selected.

After genotyping, the samples with combined Q705K in the *NLRP3* gene and C10X in the *CARD8* gene, i.e. 73 cases (71% male, 29% female; mean age: 42 years, range: 20-65 years; Figure 1, black boxes) were compared to 147 age and gender matched non-carrier controls, i.e. wild-type of both Q705K and C10X (71% male, 29% female, mean age: 42 years, range: 20-67 years; Figure 1, white box).

In addition, samples from carriers of either homozygote or heterozygote C10X *per se* (n= 47: 57.4% male, 42.6% female; mean age: 42 years, range: 19-65 years, and n=50: 58% male, 42% female; mean age: 42 years, range: 20-64 years, respectively), or from heterozygote Q705K *per se* (n=50: 74% male, 26% female; mean age: 43 years, range: 21-64 years), or from non-carrier controls (n=100: 58% male, 42% female; mean age: 42 years, range: 20-64 years) were randomly selected (Figure 1, grey boxes for cases and white box for controls). The homozygote Q705K sub-cohort contained only 2 samples that were investigated but not subjected to any statistical analysis.

The donations of blood and plasma followed the national guidelines provided by the Swedish National Board of Health and Welfare, which means that the blood and plasma donors are approved by fulfilling the following criteria: They must be healthy individuals without infectious diseases, like malaria, HIV, hepatitis or tuberculosis, or autoimmune diseases, like rheumatoid arthritis, Crohn’s disease, multiple sclerosis, neither cancer, diabetes nor coronary artery diseases. These individuals cannot be on any regular medication or have used other substances, like anabolic steroids and growth hormones, and have been asked to omit medication with glucocorticoids, antibiotics or NSAID on the day of blood donation. At the day of blood donation, the donors state and sign a Declaration of health, certifying that they are free of on-going infections and that at least 2 weeks have passed since the donors had an infectious disease or fever, 1 day since a visit with minor treatment at the dentist, and at least 6 months since any severe infections or intervention causing inflammation, such as surgery, piercing or tattooing.

### Ethics Statement

The study was conducted in accordance with the ethical guidelines of Declaration of Helsinki, and followed the ethical policy at Örebro University Hospital. The blood was collected from the study participants (i.e. healthy voluntary blood donors) after written informed consent for research use of the donated blood. The blood samples were anonymized by the Department of Transfusion Medicine at Örebro University Hospital, and the only information revealed to the study was gender and year of birth. Since the blood for research purpose was collected at the time for blood donation, no extra harm or risk was put to the donors, and since only anonymized samples were delivered to the researchers, the study did not require ethical approval according to paragraph 4 of the Swedish law (2003:460) on Ethical Conduct in Human Research.

### DNA isolation

Genomic DNA was extracted from whole blood using EZ1 DNA BLD Card in a Biorobot EZ1 (Qiagen) and the EZ1 DNA Blood Kit according to the supplier’s recommendation (Qiagen Group, Valencia, CA, USA). DNA concentration was measured in 200 randomly chosen DNA extract in NanoDrop ND-1000 (*Saveen* Werner AB, Malmö, Sweden). A mean DNA concentration value was determined and adopted on all DNA extracts, which subsequently were diluted to a final concentration of 20 ng/µL; according to the calculated mean value. DNA stock solutions were kept at 4°C between experiments.

### Genotyping by Real-Time PCR

Genetic analysis was performed for the polymorphisms C2107A in exon 3 of the *NLRP3* gene (rs35829419, which encodes Q705K), and T30A in exon 5 in the *CARD8* gene (rs2043211, which encodes C10X) [13]. The analysis was performed by a TaqMan^®^ SNP genotyping assay with 7900HT Fast Real-Time PCR system (Applied Biosystems, Foster City, CA, USA). In brief, 40 ng genomic DNA from carriers (denoted: cases) and non-carriers (denoted: controls) were amplified in a 10 µL reaction containing 5 µL 2x TaqMan^®^ Genotyping Master Mix (Applied Biosystems), 0.25 µL TaqMan^®^ SNP Genotyping assays Pre-designed primers and probes. For the genotyping, the C_ _25648615 and C_ _ 11708080_1_ genotyping mix (Applied Biosystems) were utilized. The samples were amplified according to a TaqMan^®^ standard genotyping protocol, followed by allelic discrimination analysis to evaluate the frequencies of the different alleles.

### Analysis of cytokines

To analyze the levels of IL-1β ((HSCYTO-60SK (IL-1β high sensitive), IL-33 (MPXHCYP2-62K), and IL-6, IL-8, TNF and IL-1Ra (4-plex kit MPXHCYTO-60K), commercial Milliplex Human Cytokine kits were used according to the manufacturer’s instructions (Millipore Corporation, Billerica, MA, USA). The plates were analyzed on the Luminex 200^TM^ system (MAP^TM^ technology, Austin, Texas USA). For detection of IL-18 levels, a commercially available ELISA kit was used in consistent with the manufacturer’s instructions (Code #7620 Medical & Biological Laboratory Co. LTD, Nagoya, Japan). Due to low plasma levels in some samples, it was not possible to analyze cytokine markers in all samples.

### Statistical analysis

The cytokine data experiments were performed in duplicate, and data, presented as mean, were used for statistical analysis. Normality of data sets was assessed using the Shapiro Wilk’s test. The result of the interleukin analyses were found to be non-normal distributed, why further analysis was performed with non-parametric Mann-Whitney U-test, and data were presented as median (25%-75% percentile). The results were considered significant at *p*<0.05 (Prism 5, GraphPad Software).

## Results

### Genotype and allele frequencies of the NLRP3 Q705K and CARD8 C10X genes among healthy blood donors

Genotyping was performed in 1006 human genomic DNA samples screening for the C/A polymorphism of Q705K (rs35829419) in *NLRP3* and the T/A polymorphism of C10X (rs2043211) in *CARD8* genes. The analysis was successfully performed in 1003 samples, as 3 samples were undetermined and thereby excluded. In both cases and control groups, both polymorphisms were found to be in Hardy-Weinberg Equilibrium. Details regarding the genotype and allele frequencies for the polymorphisms Q705K in the *NLRP3* gene and the C10X in the *CARD8* gene are presented in Tables 1 and 2.

**Table 1:** Genotype frequencies (%) of the Q705K (rs35829419) in the *NLRP3* gene and C10X (rs2043211) in the *CARD8* gene and in combination with each other in healthy blood donors (n=1003).

**Genotype**		***NLRP3***			
		**QQ (%)**	**QK (%)**	**KK (%)**	**Total (%)**
***CARD8***	**CC (%)**	374 (37.3)	66 (6.6)	2 (0.2)	442 (44.1)
	**CX (%)**	397 (39.6)	64 (6.4)	1 (0.1)	462 (46)
	**XX (%)**	91 (9.1)	8 (0.8)	0 (0)	99 (9.9)
	**Total (%)**	862 (85.9)	138 (13.8)	3 (0.3)	1003 (100)

**Table 2:** Allele frequencies (%) in controls (n=2006) of the polymorphism C10X (rs2043211) in the *CARD8* gene and Q705K (rs35829419) in the *NLRP3* gene.

	***NLRP3*** (n=2006)		***CARD8*** (n=2006)
**Q**	1862 (92.8)	**C**	1346 (67.1)
**K**	144 (7.2)	**X**	660 (32.9)
**Total**	2006 (100)	**Total**	2006 (100)

The frequency of a combined heterozygous genotype (QK for the *NLRP3* gene and CX for the *CARD8*) was 6.4%. A combined wild-type genotype (QQ for the *NLRP3* gene and CC for the *CARD8* gene) was found in 37.3% individuals, whereas no individual displayed a genotype that was homozygous for both polymorphisms investigated. Few individuals were found to have a combined genotype being heterozygous for QK in *NLRP3* and homozygous XX in *CARD8* (0.8%), or combined genotypes involving homozygous KK in *NLRP3* and heterozygous for CX in *CARD8* (0.1%), or homozygous KK in *NLRP3* wild-type for CC in *CARD8* (0.2%). Additional combined genotypes are presented in Table 1. Genotype frequencies in regard to gender are presented in Table S1.

### Spontaneous cytokine secretion in cases and controls

Cytokine analyses were performed in plasma from 73 individuals displaying combinations of Q705K in *NLRP3* plus C10X in *CARD8* polymorphisms (denoted: combined cases; Figure 1, black boxes). For each case, two gender and age (±1 year) matched controls were selected from healthy blood donors displaying wild-type for *NLRP3* and *CARD8* (denoted: controls; Figure 1, white box). To find out the contribution of each polymorphism *per se*, IL-1β was analyzed in plasma from individuals either carrying homozygote (n=47) or heterozygote (n=50) C10X in combination with wild-type for Q705K, and from 2 homozygote and 50 heterozygote carriers of Q705K in combination wild-type for C10X, respectively, compared to 100 non-carrier controls (Figure 1, grey boxes and white box, respectively).

**Figure 2:**
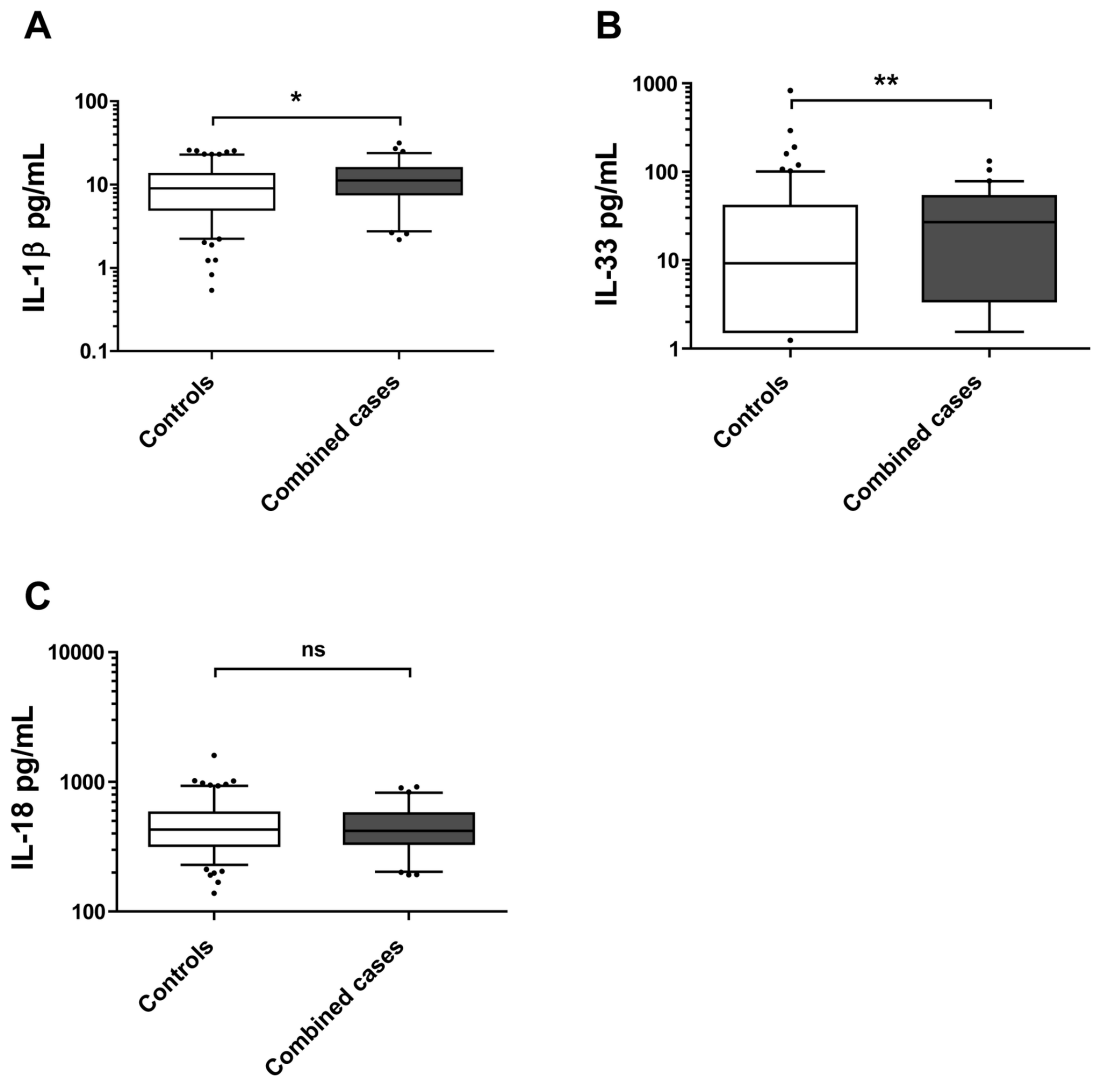
Basal cytokine levels in plasma from Combined cases and Controls. Basal levels of IL-1β (**A**), IL-18 (**B**) and IL-33 (**C**) were measured by Luminex and ELISA, respectively, in plasma from healthy blood and plasma donors. *Combined* *cases* indicate individuals carrying combined polymorphisms of *NLRP3* (Q705K) plus *CARD8* (C10X) (n=69 (A), n=71 (B), n=71 (C)), whereas *Controls* are non-carriers of these polymorphisms (i.e. wild-types) (n=140 (A), n=139 (B), n=141 (C)). Box plots display the median value and 25^th^ and 75^th^ percentile. Whiskers represent 5-95 percentiles and dots the outliners. Data were analyzed by Mann-Whitney U-test as distribution was non-normal. *p<0.05, **p<0.01 and ns = non-significant for Combined cases vs. Controls values.

**Figure 3:**
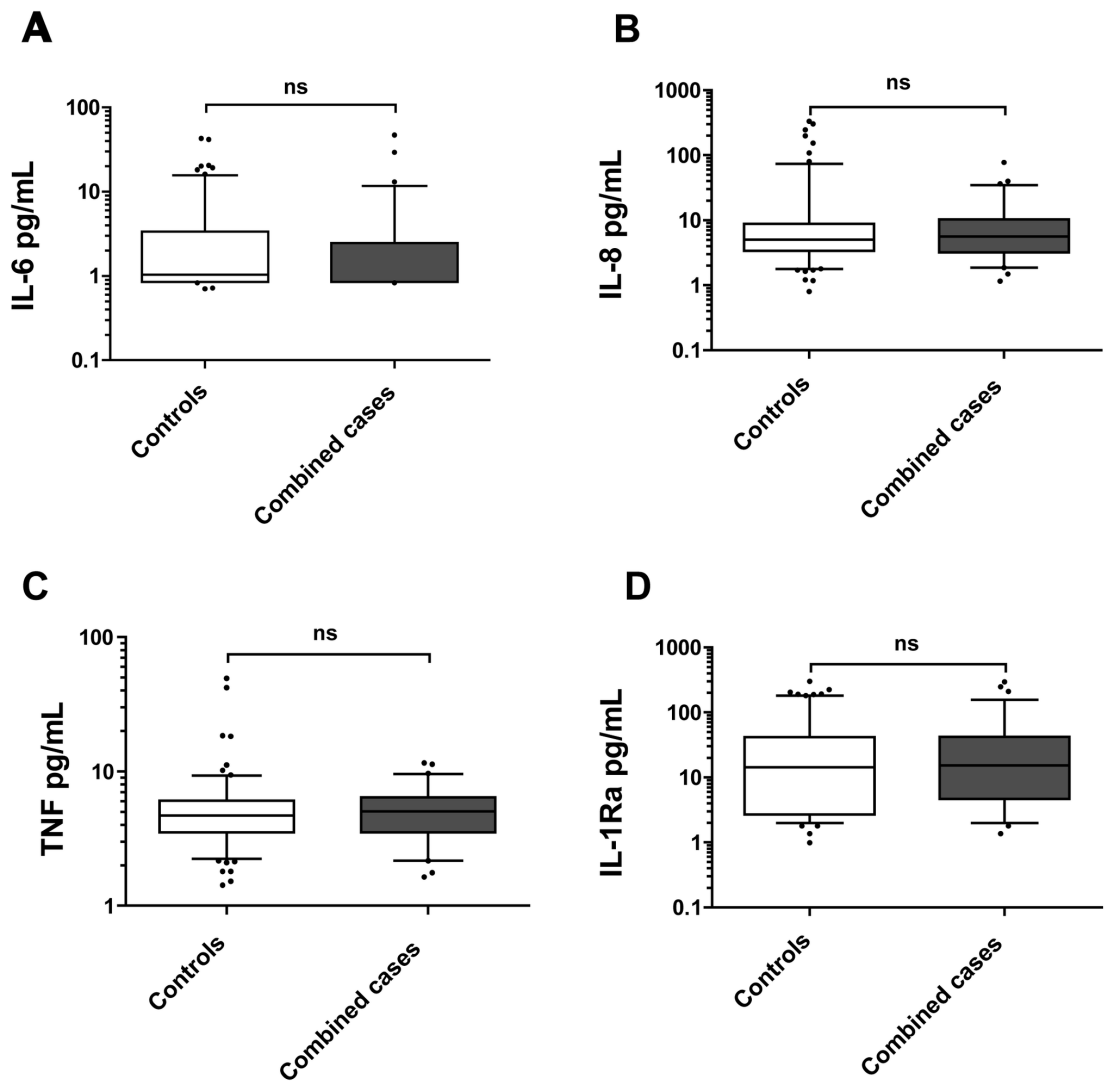
Basal cytokine levels in plasma from Combined cases and Controls. Spontaneous cytokine levels IL-6 (**A**), IL-8 (**B**), TNF (**C**) and IL-1Ra (**D**) were measured by Luminex, in plasma from healthy blood and plasma donors. *Combined* *cases* indicate individuals carrying combined polymorphisms of *NLRP3* (Q705K) and *CARD8* (C10X), whereas *Controls* are non-carriers of these polymorphisms (i.e. wild-types). (**A**) Spontaneous IL-6 levels (median values: 0.85 pg/mL (0.85-2.46) vs. 1.03 pg/mL (0.85-3.35), *p*=0.2416) comparing Combined cases (n=71) and Controls (n=143). (**B**) Spontaneous IL-8 (CXCL8) levels (median values: 5.61 pg/mL (3.2-10.25) vs. 5.05 pg/mL (3.38-8.81), *p*=0.9132) comparing Combined cases (n=71) and Controls (n=143). (**C**) Spontaneous TNF levels (median values: 5.04 pg/mL (3.51-6.42) vs. 4.69 pg/mL (3.52-6.05), *p*=0.4278) comparing Combined cases (n=70) and Controls (n=143). (**D**) Spontaneous IL-1Ra levels (median values: 15.30 pg/mL (4.65-42.12) vs. 14.29 pg/mL (2.68-41.49), *p*=0.8397) comparing Combined cases (n=71) and Controls (n=143). Box plots display the median value and 25^th^ and 75^th^ percentile. Whiskers represent 5-95 percentiles and dots the outliners. Data were analyzed by Mann-Whitney U-test as distribution was non-normal. ns = non-significant for Combined cases vs. Controls values.

**Figure 4:**
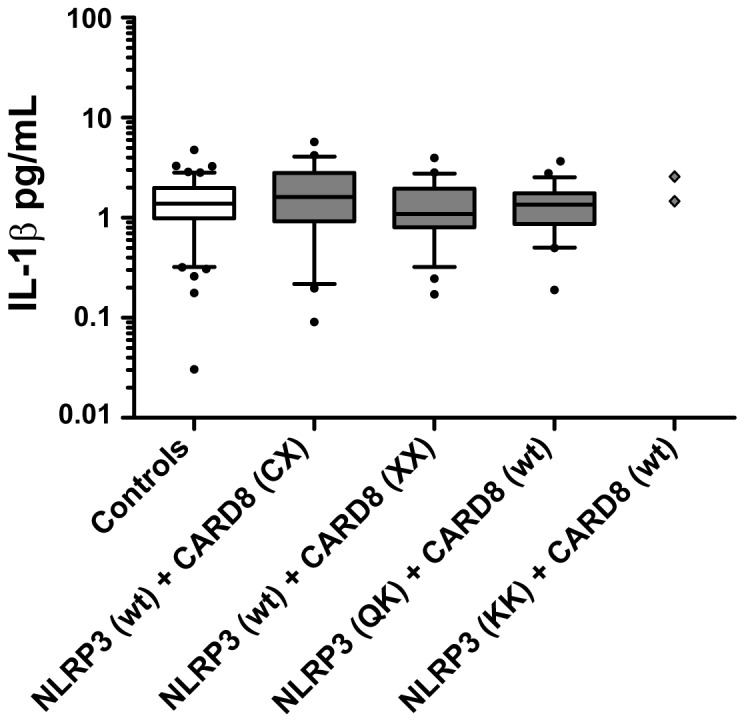
Basal IL-1β levels in plasma from Cases and Controls. Basal levels of IL-1β were measured by Luminex in plasma from healthy blood and plasma donors. *Cases* indicate individuals carrying either *NLRP3* (Q705K) heterozygote (n=50) or homozygote (n=2, ◊), or *CARD8* (C10X) heterozygote (n=50) or homozygote (n=47), whereas *Controls* are non-carriers of these polymorphisms (i.e. wild-types) (n=100). Box plots display the median value and 25^th^ and 75^th^ percentile. Whiskers represent 5-95 percentiles and dots the outliners. Data were analyzed by Mann-Whitney U-test as distribution was non-normal.

Combined cases showed higher plasma levels of IL-1β as compared to non-carrier controls (median values: 11.2 pg/mL (7.64-15.81) vs. 9.02 pg/mL (4.99-13.36), *p*=0.021; Figure 2A), as well as higher plasma levels of IL-33 (median values: 27.07 pg/mL (3.44-52.50) vs. 9.26 pg/mL (1.54-41.01), *p*=0,0094; Figure 2B). No difference was found in IL-18 levels when comparing combined cases and controls (median values: 418.5 pg/mL (334.9-570.5) vs. 428.5 pg/mL (321.9-578.5), *p*=0.93; Figure 2C). In addition, when analyzing plasma levels of cytokines synthesised down-stream of the IL-1 receptor (IL-1R), i.e. IL-6, IL-8 (CXCL8) and TNF [22], no differences were detected comparing combined cases and controls (Figure 3 A-C). This result was also found for IL-1Ra (Figure 3D). Cases carrying either Q705K or C10X *per se* were found to have similar plasma levels of IL-1β as non-carrier controls (Figure 4).

## Discussion

In the present study, 1006 healthy blood/plasma donors were screened for Q705K polymorphism in *NLRP3* and for C10X polymorphism in *CARD8*. The allele frequencies of the Q705K and C10X in the cohort were 7.2% and 32.9%, respectively, which is in accordance with previous reports in Swedish cohorts (6.5% and 34%, n=806 [13];, 6.8% and 46%, n=742 [17];), whereas the NCBI reference assembly on the European population demonstrated slightly lower allele frequencies (5.8% and 27.4%). The prevalence of combined heterozygotes, i.e. carriers of both single SNPs, was 6.4%, which is in agreement to what has previously been reported (4-6.5% [13,16,23]).

*NLRP3* was discovered a decade ago [24], while fine mapping the gene responsible for one of the auto-inflammatory diseases later on categorized under the name CAPS (“Cryopyrin-Associated Periodic Syndromes”) [25]. While these syndromes display certain distinct characteristics, they also share a number of overlapping symptoms involving recurrent fever, rashes and joint pain, as well as leukocytosis and elevated C-reactive protein (CRP) [26]. Today, more than 70 gain-of-function mutations in *NLRP3* have been found associated with CAPS. These alternations render the protein into an active conformation, resulting in unregulated amounts of IL-1β that gives rise to inflammation [27,28]. During the last years, a number of patients with CAPS-related symptoms have been found to display genetic alterations in the *NLRP3* gene; alterations also prevalent in healthy individuals [12,13]. Most patients with typical, as well as atypical CAPS, show remarkable benefit to treatment with IL-1β blockade; demonstrating a key role for this cytokine in disease pathogenesis [13,29]. In patients with CAPS-like symptoms, the Q705K polymorphism in the *NLRP3* gene in conjunction with C10X in *CARD8* has been found to correlate with increased caspase-1 activity and IL-1β secretion, as well as with dysregulated apoptosis [13,30]. These combined polymorphisms have also been implicated in several chronic inflammatory diseases [13,16-18]. Due to the abundance of these polymorphisms in the general population, it is highly relevant to study their functional significance in disease development and their impact on disease severity. The present study therefore investigates the functional status of the inflammasome in individuals of a healthy cohort carrying these polymorphisms of the NLRP3-inflammasome *per se* or combined, by detecting inflammasome-produced cytokines.

Healthy carriers of both SNPs, i.e. combined cases, showed a small but statistically significant higher level of IL-1β in plasma as compared to non-carrier wild-type controls. Whether or not this small increase actually has clinical significance needs to be further elucidated. Nevertheless, the difference in basal levels of IL-1β in plasma between patients with Q705K+C10X polymorphisms and healthy non-carrier controls has been shown to be small (5.6 vs. 2.7±0.4 pg/mL [13] and 1.6±0.06 vs. 0.4±0.1 pg/mL [30]) but does have clinical effect as the symptoms were relieved by IL-1β blockade. Plasma levels of IL-1β is subjected to an exceptional tight regulation, involving both its synthesis by caspase-1 as the inflammasome assembles, as well as its signalling that is balanced by the presence of IL-1Ra [3,31]. The difference in IL-1β between cases and controls detected in the present study is smaller than what has previously been found between patients and non-carrier controls [13,30]. In this regard, it is important to bear in mind that cases actually are healthy blood/plasma donor, which makes the finding of a small but significant increase among healthy carriers interesting in the context of disease development.

On one hand, the increased levels of IL-1β suggest that the Q705K+C10X polymorphisms may render the NLRP3-inflammasome more active at basal condition. This could provide individuals with polymorphisms in the NLRP3-inflammasome with a favourable innate immune response to infections. In line with this, patients with recurrent fever syndromes associated with increased IL-1β levels such as familial cold auto-inflammatory syndrome, the mildest form of CAPS, and Periodic Fever, Aphthous stomatitis, Pharyngitis and Adenitis (PFAPA) syndrome often have a lower frequency of upper respiratory infections (H. Hoffman, personal communication). Whether or not a slightly more active (primed) inflammasome provide a better protection against microbes needs to be further investigated. Future studies should therefore investigate the inflammasome response in carrier individuals in comparison with non-carriers upon challenging their cells *ex vivo* with various pathogens.

On the other hand, the Q705K+C10X polymorphisms leading to a hyperactive inflammasome may be detrimental to the host due to exaggerated inflammatory responses, as observed in a previously reported patient [13] or lead to sustained inflammation supported by epidemiologic data suggesting increased susceptibility to chronic inflammatory conditions, e.g. rheumatoid arthritis and Crohn’s disease [16-18]. So, what mechanisms are involved to convert an appropriate/proper inflammasome response into an exaggerated immune reaction? Functional studies using an *in vitro* model in which THP-1 cells (that have a C10X background) were transduced with the Q705K variant revealed it to be a gain-of-function variant, which resulted in a lower threshold for inflammation [20]. Therefore, we hypothesize that the Q705K+C10X polymorphism acts as low-penetrance genetic variant, that is most likely also dependent on other host related genetic factors and/or environmental exposures. This may also explain why patients with Q705K+C10X polymorphisms display heterogeneous symptoms [16-18,30].

Speaking against a clinical impact of the increased levels of IL-1β detected in combined carriers, are the data showing that IL-6, IL-8 (CXCL8) and TNF, which can be synthesised downstream of the IL-1R [22], were produced in similar concentrations in combined cases and controls. In Q705K-transduced cells, we have found the release of TNF to be a downstream event of IL-1R activation by IL-1β, whereas the increased gene expression of TNF in these cells was not an IL-1R regulated event [20]. These data suggest the involvement of other pathways or other component of the inflammasome (e.g. ASC [32,33]) to be functional in regulating the expression of IL-6, IL-8 and TNF, which could thereby explain our findings of different release-pattern of the various cytokines. The transcription factor NF-κB controls the expression of these pro-inflammatory cytokines, and, IL-18, among other signalling molecules, has been shown to activate NF-κB [34].

IL-1Ra was also produced in similar concentrations between cases and controls. IL-Ra is under normal conditions produced in balance with IL-1β (reviewed in [31]:). Nevertheless, because of the spare receptor effect, 100-fold or greater levels of IL-1Ra over IL-1β are necessary to functionally inhibit the biologic effects of IL-1β on target cells [35]. Thus, IL-1Ra must be produced abundantly to block the effects of IL-1β, and a difference between healthy carriers and non-carriers, regarding this cytokine, may thereby not be applicable.

In contrast to IL-1β production, IL-18 was found to be produced in equal amount in healthy combined carriers and non-carriers. Both these cytokines are produced via caspase-1 action but unlike IL-1β, IL-18 is constitutively expressed in human PBMCs and other cell types and thus does not require a priming process for precursor induction [36], which might explain the different expression of IL-1β vs. IL-18 in plasma from carriers and non-carriers. Interestingly, plasma levels of IL-33, a novel cytokine of the IL-1 cytokine family, were significantly higher in combined cases compared to controls. IL-33 has also been suggested as a substrate for caspase-1 and thereby dependent on a proper inflammasome assembly. However, in contrast to IL-1β and IL-18, IL-33 display bioactivity in its full-length form and the caspase-1 processing results rather in its inactivation [6]. Even so, our findings of increased plasma levels of IL-1β and IL-33 indicate the presence of an inflammasome with increased basal activity in carrier individuals. Additional pathways have however been suggested in the release of these cytokines (reviewed in [37-39]:), which could explain the divergent production of IL-1β and IL-33 versus IL-18 detected in our study of combined carriers of the Q705K+C10X polymorphisms vs. non-carriers. Also, the situation and/or cell type in which the inflammasome is activated could influence the synthesis of IL-1β and/or IL-18, as IL-18 secretion from microglia has recently been shown to occur independently of inflammasome action, whereas the production of IL-1β was partly dependent of caspase-1 in these cells [40]. More studies are required to find out the mechanisms during which inflammasome-dependent cytokines are produced and to understand if and how genetic variations in the *NLRP3*/*CARD8* genes modulate the inflammasome action of these cytokines.

The Q705K polymorphism renders NLRP3 into a gain-of-function phenotype [20], which results in a lower threshold for activation, whereas the C10X polymorphism results in significantly decreased expression of CARD8 [21,41]; and thus the loss of CARD8-mediated inhibition of caspase-1. These data suggest each polymorphism *per se* to create inflammasomes with a lower threshold for stimulation. Our data do however not find any increased IL-1β production in carriers of either C10X or Q705K; suggesting that each polymorphism *per se* does not influence the basal activity of the inflammasomes in healthy individuals. On the one hand, C10X, as well as Q705K, has been found associated with susceptibility and severity of a number of diseases [15,23,42-44]; indicating an add-on contribution of other host related genetic factors and/or environmental exposures for disease development. On the other hand, it has been speculated that C10X-carriers have a more robust inflammatory response, and thus, better survive under conditions with higher infectious-disease burden [21]. Taken together, these data highlights the existence of two sides of the same coin in which the NLRP3 polymorphisms under certain circumstances provide beneficial effects, whereas during others results in harmful conditions.

In conclusion, we find significantly higher levels of IL-1β in plasma from healthy individuals carrying combined polymorphisms in *NLRP3* and *CARD8* genes compared to non-carriers; data that indicate the presence of inflammasomes with higher activation state in leukocytes from these individuals. This may provide carrier individuals with a more favourable innate immune response against infections [21], but could also represent the mechanisms by which the inflammatory loop is triggered into a long-term inflammatory phenotype [13,16-19,30].

## Supporting Information

Table S1 **Combined genotype frequencies (%) in healthy male (n=636) and females (n=367), of the Q705K (rs35829419) in the NLRP3 gene and C10X (rs2043211) in the CARD8 gene.**
(DOCX)
