# Supplementary material for: Cytokine Profile in a Cohort of Healthy Blood Donors Carrying Polymorphisms in Genes Encoding the NLRP3 Inflammasome
Source: PLoS One. 2013 Oct 3;8(10):e75457. doi: 10.1371/journal.pone.0075457 (PMC3789710; doi:10.1371/journal.pone.0075457)
Supplement: Table S1 — Combined genotype frequencies (%) in healthy male (n=636) and females (n=367), of the Q705K (rs35829419) in the NLRP3 gene and C10X (rs2043211) in the CARD8 gene. (DOCX) [file pone.0075457.s001.docx]

**Table S1**

| **Genotype** |  | ***NLRP3*** | | | | | |
| --- | --- | --- | --- | --- | --- | --- | --- |
|  |  | **QQ** | | **QK** | | **KK** | |
| ***CARD8*** |  | Male | Female | Male | Female | Male | Female |
|  | **CC** | 239 (37.6) | 135 (36.8) | 46 (7.2) | 20 (5.4) | 0 (0) | 2 (0.5) |
|  | **CX** | 243 (38.2) | 154 (42) | 48 (7.5) | 16 (4.4) | 0 (0) | 1 (0.3) |
|  | **XX** | 56 (8.8) | 35 (9.5) | 4 (0.6) | 4 (1.1) | 0 (0) | 0 (0) |
